# Marine litter plastics and microplastics and their toxic chemicals components: the need for urgent preventive measures

**DOI:** 10.1186/s12302-018-0139-z

**Published:** 2018-04-18

**Authors:** Frederic Gallo, Cristina Fossi, Roland Weber, David Santillo, Joao Sousa, Imogen Ingram, Angel Nadal, Dolores Romano

**Affiliations:** 1 SCP/RAC, Barcelona Convention for the Protection of the Marine Environment and the Coastal Region of the Mediterranean, Stockholm Convention Regional Activity Centre in Spain, Barcelona, Spain; 2 University of Siena, Siena, Italy; 3 POPs Environmental Consulting, Schwäbisch Gmünd, Germany; 4 Greenpeace Research Laboratories, Exeter, UK; 5 Global Marine and Polar Programme, International Union for Conservation of Nature (IUCN), Gland, Switzerland; 6 International POPs Elimination Network (IPEN), Rarotonga, Cook Islands; 7 Endocrine Society EDC Advisory Group Chair, Miguel Hernandez University of Elx, Alacant, Spain; 8 Independent Consultant, Zaragoza, Spain

**Keywords:** Plastic waste, Microplastics, Nanoplastics, Endocrine disruptors, Persistent organic pollutants, Marine biodiversity, Stockholm Convention, Basel Convention, Prevention measures, Food security

## Abstract

Persistent plastics, with an estimated lifetime for degradation of hundreds of years in marine conditions, can break up into micro- and nanoplastics over shorter timescales, thus facilitating their uptake by marine biota throughout the food chain. These polymers may contain chemical additives and contaminants, including some known endocrine disruptors that may be harmful at extremely low concentrations for marine biota, thus posing potential risks to marine ecosystems, biodiversity and food availability. Although there is still need to carry out focused scientific research to fill the knowledge gaps about the impacts of plastic litter in the marine environment (Wagner et al. in Environ Sci Eur 26:9, 2014), the food chain and human health, existing scientific evidence and concerns are already sufficient to support actions by the scientific, industry, policy and civil society communities to curb the ongoing flow of plastics and the toxic chemicals they contain into the marine environment. Without immediate strong preventive measures, the environmental impacts and the economic costs are set only to become worse, even in the short term. Continued increases in plastic production and consumption, combined with wasteful uses, inefficient waste collection infrastructures and insufficient waste management facilities, especially in developing countries, mean that even achieving already established objectives for reductions in marine litter remains a huge challenge, and one unlikely to be met without a fundamental rethink of the ways in which we consume plastics. This document was prepared by a working group of Regional Centres of the Stockholm and Basel Conventions and related colleagues intended to be a background document for discussion in the 2017 Conference of the Parties (COP) of the Basel Convention on hazardous wastes and the Stockholm Convention on persistent organic pollutants (POPs). The COP finally approved that the issue of plastic waste could be dealt by its Regional Centres and consistently report their activities on the matter to next COP’s meetings.

## Background—situation on plastic and related chemical contamination and impacts

### Plastics in the ocean: sources, volumes, trends

Plastic marine litter is a mixture of macromolecules (polymers)^1^ and chemicals, its size ranging from several metres to few nanometres. It comprises such diverse items as fishing gear, agricultural plastics, bottles, bags, food packaging, taps, lids, straws, cigarette butts, industrial pellets, and cosmetic microbeads, and the fragmentation debris coming from the weathering of all of them. It has become ubiquitous in all marine compartments, occurring on beaches; on the seabed; within sediments; in the water column and floating on the sea surface. The quantity observed floating in the open ocean represents only a fraction of the total input: over two-thirds of plastic litter ends up on the seabed with half of the remainder washed up in beaches and the other half floating on or under the surface, so quantifying only floating plastic debris seriously underestimates the amounts of plastics in the oceans [1]. There are major concentration patches of floating plastics in all the five big ocean gyres, and there is evidence that even the polar areas are acting as additional global sinks of floating plastics [2].

**Fig. 1:**
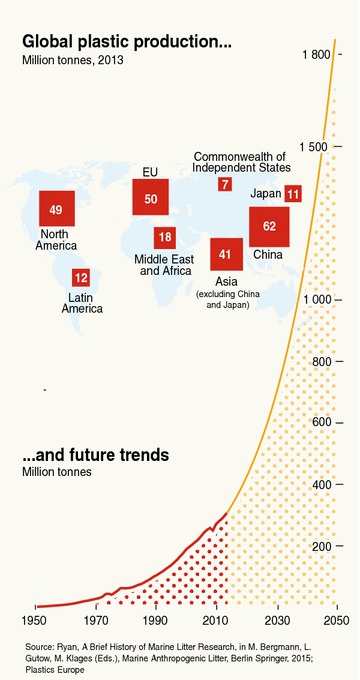
Global plastic production and future trends (Source: UNEP. Marine plastic debris and microplastics—Global lessons and research to inspire action and guide policy change [3]); Marine Litter Vital Graphics- www.grida.no. Cartographer: Maphoto/Riccardo Pravettoni

The global production of plastics is following a clear exponential trend since the beginning of mass plastic consumption and production in the 1950s, and from a global production of 311 million tonnes in 2014, it is projected to reach around 1800 million tonnes in 2050 (Fig. 1) [3]. The quantities of plastics leaking to the oceans on a global scale are largely unknown. Reliable quantitative estimations of input loads, sources and originating sectors represent a significant knowledge gap, but it is suggested that, every year, almost 8 million tonnes of plastic leak to the ocean. It is estimated that the ocean may already contain over 150 million tonnes of plastic [4], of which around 250,000 tonnes, fragmented into 5 trillion plastic pieces, may be floating at the oceans’ surface [5]. It has also been estimated that the global quantity of plastic in the ocean will nearly double to 250 million tonnes by 2025 [6],^2^ which likely also represents a pollutant load of millions of tonnes of chemical additives.

It is estimated that, on average, around 80–90% of ocean plastic comes from land-based sources, including via rivers, with a smaller proportion arising from ocean-based sources such as fisheries, aquaculture and commercial cruise or private ships. Of that 80%, three quarters is estimated to arise as a result of the lack of efficient collection schemes and proper waste management facilities in the municipalities in many countries, with the remainder entering the marine environment from careless littering and leaks from within the waste management system itself (such as urban drains).^3^

In addition to the detrimental consequences that ingestion of plastics by marine biota may entail [8–10], worrying environmental consequences of marine litter also stem from microplastics (less than 5 mm in diameter) and nanoplastics (less than 100 nm in at least one of its dimensions), which could potentially affect marine biota both from their physical nature if ingested and by transfer of chemicals associated with them, including persistent organic pollutants (POPs) and endocrine disruptor chemicals (EDCs). Most micro- and nanoplastics originate from the degradation of macroplastics through different pathways, i.e. photodegradation and other weathering processes of plastics that have leaked into the sea [1], e.g. bags, bottles, lids, food packaging, etc.; from plastic pellets lost into the environment during production or freight process; or from textile fibres coming from washing machine runoff^4^ [3, 11]. They may also be present as deliberately manufactured plastic microbeads used as scrubbing agents or for other purposes that can be found in some personal care and cosmetic products. It has been estimated that in the USA alone, even considering that all sewage is connected to tertiary waste water treatment plants (WWTP), and assuming a 99% efficiency of the sedimentation process, around 8 trillion microbeads may nevertheless be released into aquatic habitats every day. Furthermore, as the sludge of the WWTPs may subsequently be applied as fertilizer, part of the remaining 800 trillion microbeads may enter into soils and aquatic habitats via runoff [12].^5^ Some wildlife may also contribute to the overall burden of microplastics when they ingest larger pieces of plastic which are then broken up into smaller pieces in their guts and lost back into the environment in form of microplastics. For example, fulmars (*Fulmarus glacialis*), a type of seabird, alone are estimated to reshape and redistribute annually about 6 tonnes of microplastics [13].

Uptake of microplastics through different mechanisms has been demonstrated in more than 100 marine species, from zooplankton to whales, including mussels, crabs, fish, planktivorous sharks, sea reptiles and seabirds. In some species, ingestion is reported in over 80% of individuals in sampled populations.^6^ Organisms can ingest microplastics as food, whether unintentionally capturing them while filter- or deposit-feeding or mistaking them for prey when foraging, or even by ingesting prey containing microplastics, i.e. trophic transfer [15]. In some species, microplastics can be taken into the body when they become entrapped by gill structures [16, 17]. Microplastics and nanoplastics fall well within the size range of the staple phytoplankton diet of many zooplankton species, such as the Pacific krill. Fossi et al. [18] found that 56% of surface neustonic/planktonic samples from the Mediterranean Sea contained microplastic particles.

Microalgae attached to microplastics are assumed to be more easily captured by filter feeders than free microplastics in the water column [15]. After microplastics are assimilated into the organism they accumulate in the gut, translocate into other tissues or are excreted, depending on the size, shape and composition of the particles. For example, fish fed with langoustines (*Nephrops norvegicus)* containing polypropylene filaments were found to ingest but not to excrete the microplastic strands, further corroborating the potential for trophic transfer and ecological impacts [14, 19, 20].

Uncertainties remain regarding the extent of harm caused to marine species directly by ingestion of microplastics, and over the contribution they make to overall exposures to hazardous chemicals. Some studies report little or no physical or chemical harm to marine biota [21], while others including the use of thermodynamic approach^7^ and the simulation of physiological conditions in the gut, suggest that chemicals in plastics might be released to organisms after ingestion [22–25]. In mussels, *Mytilus galloprovincialis*, exposed to microplastics (polyethylene and polystyrene) contaminated with polyaromatic hydrocarbons, marked bioaccumulation of these chemicals was recorded in both digestive gland and gills [26]; similarly in tidal flat organisms such as lugworms, *Arenicola marina*, exposed to microplastics with adsorbed pollutants (nonylphenol and phenanthrene) and additive chemicals (Triclosan and PBDE-47) [24]. Endocytosis^8^ of plastic nanoparticles can also result in adverse toxic endpoints [1, 19].

Microplastics move with currents, wave action and wind conditions, and can be found throughout all marine compartments. Modelling the dynamics and fate of micro- and nanoplastics in the marine environment is a complex and uncertain task, since particles initially at the sea surface can sink to sediments, accelerated by biofouling, ageing, etc., while those already in sediments can potentially become remobilized to the water column by bioturbation, resuspension or hydrodynamic conditions and translocation by marine organisms [15]. It is remarkable that benthic microplastics are far more widespread than previously assumed, with accumulation trends matching the increasing production of plastics worldwide [1, 15, 20].

In the Mediterranean Sea, marine litter has become a critical issue, as this is a region known to be accumulating a high concentration of plastics [27–29]. This is due to interaction of a number of factors, including the hydrodynamics of this semi-closed sea (from which outflow mainly occurs through deep water currents), combined with a lack or deficit of environmentally sound urban waste management and proper and efficient collection systems of much of the waste generated in many of its riparian countries and heavily populated coastal areas.

Other areas of particular concern include mid-ocean islands close to gyres and the Small Island Developing States (SIDS), where the situation has been depicted as “waste disaster” [30]. In addition to the challenge of marine litter, these States face serious deficiencies in basic waste management capabilities, due mainly to small and sparse populations with limited potential economies of scale. There is also a shortage of land for sanitary landfill, with waste often being disposed of casually by burial, burning or discard into the surrounding land and sea. Furthermore, consumption patterns are changing over time, with an increasing number of tourists and more plastic waste being generated overall. The state and pace of economic and social development in these small and remote countries, faced with growing populations and increasing urbanisation and with limits to infrastructure and to both human and natural resources, make combatting this growing threat to their supporting ecosystems and means of life extremely challenging [3].

At a global level, UNEP has estimated the economic impact of marine plastics (excluding microplastics), including losses incurred by fisheries and tourism due to plastic littering, as well as beach clean-up costs, at around $13 billion per year [31].

### Chemicals (POPs and EDCs) in marine litter plastics: fate in the marine environment

Besides the adverse physiological effects to marine organisms that arise from ingestion of pieces of plastic, plastics in the marine environment may also pose an additional chemical hazard, especially those containing known or suspected endocrine disrupting chemicals as additives or contaminants. Although plastics will not be the only route by which marine species are exposed to hazardous chemicals, existing evidence supports mounting concern in the scientific community that plastics may nonetheless make a significant contribution to exposures to complex mixtures of chemical contaminants [14, 18, 20, 26, 29, 32–38, 69, 39] The chemicals found in plastic marine litter can be classified in the following four categories of origin:

- Chemicals intentionally added during the production process (additives such as flame retardants, plasticizers, antioxidants, UV stabilisers, and pigments);
- Unintentional chemicals coming from the production processes, including monomers (e.g. vinyl chloride, BPA, etc.)^9^—which may also originate from UV radiation onto the plastic waste—and catalysts, normally present in traces (ppm);
- Chemicals coming from the recycling of plastic waste^10^; and finally,
- Hydrophobic chemicals adsorbed from environmental pollution onto the surface of the plastics.^11^

Whatever their origin, such substances may be directly released from plastics when they reach the guts of marine species, and may otherwise leach to the marine environment when the plastic weathers, at a rate depending of factors such as the nature and strength of the bound between additive and polymer (reactively bonded compounds requiring more energy), pore diameter, molecular weight of the additive, temperature, pressure, and biofouling.

Chemicals with endocrine disrupting properties are a major concern for the marine environment. A compilation of lists of chemicals recognised as Endocrine Disrupting Chemicals (EDCs) or suggested as Potential EDCs has been developed by the International Panel on Chemical Pollution (IPCP) [42]. The SINList^12^, developed by ChemSec, compiles those chemicals with most urgent action needed.

In more general terms, experimental research on animals shows that low-level, non-linear exposures to endocrine disruptor chemicals (EDCs) lead to both transient and permanent changes to endocrine systems, as EDCs can mimic, compete with, or disrupt the synthesis of endogenous hormones [20, 43, 44]. This results in impaired reproduction and consequent low birth rates and potential loss of biodiversity, thyroid function, and metabolism, and increased incidence and progression of hormone-sensitive cancers [45]. The research suggests that embryo and developmental periods are critical-sensitive periods to EDCs.^13^ EDCs may cause effects in cellular and/or animal models at extremely low concentrations [45].

Some of those intentional chemical additives in plastics with toxic and endocrine disrupting properties might be present at levels of 1000–500,000 mg/kg (ppm). This is the case of polybrominated diphenyl ethers (PBDEs) used as flame retardants in plastics, polyurethane foams and textiles; tetrabromobisphenol A (TBBPA)^14^ [40], used as flame retardant in epoxy, vinyl esters and polycarbonate resins; or hexabromocyclododecane (HBCDD), used in polystyrene foam (EPS/XPS) or di-2-ethylhexyl phthalate (DEHP) in PVC. It is also recognised that such chemicals can be found as particularly prominent contaminants in marine species collected from areas in which flame retardant-treated plastics are in use. For example, elevated HBCDD levels were found in oysters from aquaculture farms at which EPS/XPS buoys containing HBCDD were present [46]. The observation that high levels of the y-HBCDD isomer, which dominates commercial mixtures of this flame retardant [47, 48], can be detected in fish in some European waters [49], indicates that direct exposure to technical HBCDD present in the polymer matrix can also be a relevant exposure pathway for fish, as well as the wider environmental exposure to the more stable α-HBCDD.

Further evidence that some POP chemicals are transferred to animal tissues directly from ingested plastic rather than from polluted prey, for example, arises from a study by Tanaka et al. [23] on short-tailed shearwaters that frequently ingest plastics that they mistake for food. These researchers focused on the presence of specific congeners of PBDEs present in the plastic but not commonly found in their prey (pelagic fish), confirming the presence of those congeners in both the fatty tissues of the birds and in the plastics found in their stomachs.

Other plastic additives of concern in the marine environment include chlorinated paraffins^15^ [50] added as flame retardants; polychlorinated biphenyls (PCBs) and polychlorinated naphthalenes (PCNs) included in PVC coatings/paints, and sometimes released as fine particles from abrasive blasting from, e.g. bridges into waters in tonnes scale^16^ [51, 52]; and per- and polyfluorinated compounds (PFCs)^17^ [53, 54]. Fluorinated polymers containing perfluorooctanesulfonic acid (PFOS) and perfluorooctanoic acid (PFOA) precursors used in some textile fibres and in paper and paperboard articles (i.e. fast-food packaging and paper plates, cups, etc.) to provide grease and water resistance [55], can become microplastics/fibres in the aquatic environment and release PFOS when degrading or ingested^18^ [56].

Other chemicals of concern include plastic additives with known or suspected endocrine disrupting properties, including alkylphenols (octylphenol and nonylphenol) used mainly as antioxidants, bisphenol A (BPA) present in polycarbonate plastics as trace monomer, phthalate esters [e.g. di(2-ethylhexyl) phthalate (DEHP), diisodecyl phthalate (DIDP), diisononyl phthalate (DINP) and butyl benzyl phthalate (BPP)], widely used as plasticizers in proportions up to 60% of the weight of a plastic to increase properties such as flexibility, transparency or longevity, and organotin compounds (based on methyl, butyl or octyl groups, such as tributyltin^19^) used as stabilizing additives in some PVC polymers. For example, Takada et al. [57] and Hirai et al. [58] analysed a wide range of chemicals in marine plastics collected from urban and remote beaches and open oceans, including theoretically “non-persistent” additives such as alkylphenols, i.e. nonylphenol, octylphenol, and BPA, which were detected in concentrations ranging from ng/g to μg/g in polyethylene and polypropylene debris.^20^ Moreover, a significant correlation has been demonstrated [18, 60] among seven different phthalate esters (phthalates or PAEs) present in samples taken in the same area of microplastics, plankton and bubbler samples of different cetacean species.^21^

Some of these chemicals with endocrine disruptor properties may not qualify as “persistent” under the strict criteria of the Stockholm Convention, which requires in the screening criteria of its Annex D evidence of its half-life in water, soil, sediments and air. Nevertheless, when present in a polymeric matrix in marine conditions, they may be potentially as harmful as officially recognised POPs in terms of behaviour and consequences in the marine environment, as their presence is ‘topped-up’ by the continuous flow of “fresh” plastic waste in river discharges, urban runoff and waste water and associated sediments [41, 61]. Their adsorption to microplastics, combined with the harsher environmental conditions of low temperature and salinity, combined also with low light and low oxygen content in subsurface waters and sediments, may also enhance their persistence in marine systems and their mobility and fluxes through all the compartments of the marine environment [15]. Sorption of contaminants in nanopores of plastics may further inhibit contaminant biodegradation [62]. Taking into account also that is very difficult or even impossible to establish a threshold of toxicity for many EDCs, as low dose effects and non-monotonic dose responses (NMDR) are common [44], the overall result would be that those substances in plastics in the marine environment may through their widespread and pervasive distribution, present equivalent levels of concerns to those of recognised POPs. In this regard, such characteristics and evidence would allow equating EDCs in marine plastic waste with the defining properties of a POP under the Stockholm Convention. This is further discussed in “Contribution from the Stockholm Convention on persistent organic pollutants”, on potential measures for the consideration of the Stockholm Convention.

It should be noted that recycled plastic/polymers can also carry a high content of toxic chemicals carried over from their source plastics, and may also therefore contribute to chemical exposure of the marine environment^22^ when they reach the ocean. The fact that much of the plastic waste collected for recycling is exported to countries with low legal requirements or technical capabilities on the control of the different types and concentrations of hazardous substances contained in the plastics^23^ is an added source of concern, as the concentration of those toxic chemicals may increase in the recycled products.

With regard to the pollutants present in sea water and adsorbed onto the plastic surface, it has been estimated that fluxes of PCBs, PBDEs and PFOA to the Arctic caused by plastic debris was in the order of four to six times smaller than fluxes caused by atmospheric or seawater currents [63]. It is important to keep in mind, however, that the significance of pollutant transport routes does not only depend on the absolute amount of pollutants, but also on their impact from direct plastic ingestion and bioaccumulation in food chains [40]. In this regard, a qualitative distinction has to be made between microplastics and nanoplastics:

In microplastics, the adsorption of pollutants has been experimentally demonstrated from virgin plastic pellets in seawater, which implies that plastics constitute both a transport medium and a potential source of toxic chemicals in the marine environment [22, 58, 59]. The mechanisms of concentration of these chemicals is a complex issue depending of multiple variables including hydrophobicity of the pollutant, type of polymer, age of the plastic, water, temperature, pressure, presence of biofouling on the plastic surface, and salinity. It is without doubt that other media present in the oceans, including natural sediments and the sorbent organic matter (SOM)—composed of suspended organic particulates, black carbon and natural diet and planktonic species—also have the capacity to adsorb hydrophobic organic chemicals (HOCs), such that ingestion of plastics will not be the only source of exposure to such chemical agents. Indeed, on average the fraction of HOCs adsorbed to marine plastics appears to be statistically smaller when compared to that adsorbed fraction in other media in the ocean, such that chemical exposure of marine biota might be dominated by those other matrices [64]. Nonetheless, for certain chemical groups and/or specific local conditions with high concentration of plastic matter, the importance of contaminant transfer from plastics may well be of quantitative significance.

In nanoplastics, the high surface area may present exceptionally strong sorption affinities for pollutants, thus changing the exposure and risk to these chemicals [65], and further increasing their significance as contributors to overall chemical exposure. In this regard, Koelmans et al. [66] affirm that: *“because of the surface effect, it may be possible that* nanoplastics *retain organic toxic chemicals or heavy metals at higher concentrations than microplastics, thus leading to a fugacity gradient to organism tissue once ingested. If* nanoplastics *are capable of permeating membranes, passing cell walls, translocate and/or reside in epithelial tissues for prolonged times, the combination of particle and chemical toxicity may yield unforeseen risks”* Velzeboer et al. [65] affirm that: *“Nanoplastics have been shown to pass through the chorion of fish eggs and have been shown to move directly from the digestive tract of mussels into their circulatory system. This implies that occurrence of HOC contaminated* nanoplastics *in the environment may potentially enhance uptake”.*

Unfortunately, there are currently no sufficiently developed analytical methods adequate to detect and quantify nanoplastics in the environment or food chain [67], let alone to analyse their chemical signature in detail.

### Potential impacts on marine biodiversity

Chronic exposure simply to the physical presence of microplastics has been linked to effects on populations, including the negative influence of micro- and nanoplastics on survival and mortality of different species of zooplankton, which represent a critical energy source in the marine environment [68], or the reduced growth of offspring and reduced survival and fecundity compared to control organisms in crustaceans [10]. The Joint Research Centre of the EC [9] concluded that there is experimental evidence of negative physical/mechanical impacts from ingestion of plastic on the condition, reproductive capacity and survival of individual marine organisms. However, the evidence is restricted to laboratory experiments with organisms from lower trophic levels. These findings imply evidence of harm in natural populations, but quantifying the extent of this harm would be extremely challenging and the extent of harm caused by ingestion is likely to be underestimated, because necropsies have to be carried out.

With regard to the chemical transfer of chemicals from plastics, there is still need of more studies for reliable estimates to be made as to the contribution to EDC exposure of marine species arising from microplastic or nanoplastics uptake, and this is a serious knowledge gap. There is already some scientific evidence suggestive of endocrine disruptor activity relating to the intake of chemicals associated with microplastics via the filter-feeding mechanisms of animals like mussels or baleen whales [18], or via the magnifying effect of the food chain in top predators such as the swordfish [69]. Although in these studies mentioned it could be questioned what the main source of phthalates is—water pollution, microplastics and/or food chain—, the most plausible thesis is that water is not the main source of the pollution: phthalates in water are found in high concentrations only in coastal environments. In the case of the baleen whales, phthalate concentrations were very high in the microplastic and krill to which the animals were exposed, while not being detected in the water, though the relative contributions of krill and microplastics to overall phthalate exposure have yet to be determined.

While it is true that the transfer of persistent organic pollutants such as PCBs to aquatic organisms from microplastic in the diet is likely a small contribution compared to other natural pathways of exposure [70], this would not be the case for non-persistent pollutants such as some EDCs which are found in greater concentrations in microplastics than in surrounding seawater or sediments.

Widely used plasticizers with endocrine disrupting properties, e.g. dibutyl phthalate, dimethyl phthalate, butyl benzyl phthalate, or plastic monomers such as Bisphenol A (BPA), can affect both development and reproduction in marine species: effect concentrations of plasticizers in laboratory experiments in some sensitive species such as molluscs, crustaceans and amphibians (including disturbance in spermatogenesis in fish) coincide with measured environmental concentrations in the low nanogram/litre to microgram/litre range. It should be remarked that there are still basic knowledge gaps, including the long-term exposures to environmentally relevant concentrations and their ecotoxicity when part of complex mixtures [61]. Other EDCs, such as alkylphenols, have the capacity to derail male reproductive development leading to feminisation or demasculinization of the male form in fish and altered sex in molluscs. Others, such as tin-containing plastic stabilisers, elicit immunological disorders in fishes and induce imposex in gastropods [71].

### Potential impacts from marine plastics on human health

Although there are no current scientific studies correlating the direct consumption of fish or shellfish contaminated with microplastics containing or polluted with EDCs and the consequent endocrine disruption effects on human health, this is perhaps not surprising given the complexity of the issue [72, 73]. One of the conclusions of the recent report of FAO on food safety [67] is that basic toxicological data on the consumption of micro and nanoplastics in humans for a food risk safety assessment are essential lacking: the available data of toxicokinetics only include absorption and distribution, whereas no information is available on metabolism and little on excretion. It is not known whether ingested microplastics can be degraded into nanoplastics, and no data are available on the potential impact that cooking and/or processing seafood at high temperature may have on the toxicity of microplastics.

According to EFSA [74], a worst case estimate of exposure to microplastics after consumption of a portion of mussels (225 g) would be 7 μg of plastics. Based on this estimate and considering the highest concentrations of additives or contaminants reported in microplastics, and assuming complete release from microplastics, the microplastics will have a negligible effect on the total dietary exposure to persistent, bioaccumulative and toxic chemicals (PBT) and plastic additives, e.g. in the case of bisphenol A (BPA), this would represent a contribution of less than 0.2% of the estimated dietary exposure to this compound in an adult of 70 kg.

With regard to existing evidence on the consequences of the uptake of micro- and nanoplastics by humans, medical literature on impact of micro- and nanoplastics originating from inhalation or released from wear debris from plastic prosthetic implants shows diverse effects varying from DNA damage, changes in gene and protein expression, cell clotting, necrosis, apoptosis, proliferation and loss of cell viability, oxidative stress, increased Ca ions, inflammation and bone osteolysis, to lesions in organs [67].

However, at this time the uncertainties surrounding potential health impacts remain high, and the data gaps, very large, including a lack of knowledge on the role and hazards of nanoplastics, potentially the most hazardous area of marine plastics [66, 75]. Given the unavoidable increase in the coming decades of micro- and nanoplastics in the marine environment due to the weathering and fragmentation of already existing ‘stocks’ of marine macroplastics as well as future inputs, there is an urgency in better resolving the nature and scale of possible health effects, and in the meantime at least, to apply the precautionary principle.^24^ Until the weight of the scientific evidence is more conclusive regarding the risk that diets rich in small fish in whole (i.e. including the guts), or in bivalves and crustaceans containing microplastics or nanoplastics in significant quantities, could affect human endocrine systems—especially during embryo and infancy stages—, or induce hepatic stress or other related health affections, it would seem wise to assume that measures that can limit or avoid intakes of microplastics would be an appropriate and important priority for public policy.

Further scientific research is needed with urgency on the potential impacts to endocrine systems and overall human health, especially on developing stages, by the direct or indirect ingestion of marine micro- and nanoplastics.

### Potential impacts on food safety and availability and economic activity

Without immediate action, the environmental impacts and the economic costs is due to increase: as mentioned in “Plastics in the ocean: sources, volumes, trends”, more than a hundred million tonnes of plastics are estimated to have been dumped already to the oceans, and projections in plastic production and consumption indicate that plastic waste inputs in the sea may have an exponential increase if no urgent actions are taken [6]: on average, plastic consumption reached 100 kg per person per year in Western Europe and North America, and 20 kg in Asia [76], and these figures are expected to grow rapidly in populated developing countries as urban population increases and urban dwellers must purchase all of their—plastic packaged—food and beverage (see Fig. 1).

As stated before, EDCs introduced via plastics may already be affecting marine biodiversity, raising additional concerns about food safety and security in a near future. Perhaps the most important source of dietary exposure of humans to microplastics at present is via filter-feeding shellfish, which retain particles from suspension on their gills for subsequent ingestion, and thus they are directly exposed to micro- and nanoplastics via the water column. There is ample evidence of the ingestion of microplastics by bivalves [26], e.g. nine of the most commercially popular species of bivalves purchased from a fishing market in Shanghai were found to be contaminated with microplastics. Based on the abundances observed, it was estimated that Chinese shellfish consumers could be exposed to 100,000 s of microplastics each year [23, 77].

In the case that marine biodiversity and food safety and availability are affected, this would represent a serious economic impact at global level, especially in countries/islands where fish is a staple food, by exacerbating poverty [41, 78, 79] in a context of climate change and growing competition for natural resources. Fish contributes, or exceeds, 50% of total animal protein intake in some Small Island Developing States, as well as in Bangladesh, Cambodia, Ghana, Indonesia, Sierra Leone and Sri Lanka [80]. It is estimated that fish, bivalves and crustaceans provide more than 3.2 billion people with almost 20% of their average per capita intake of animal protein, and 5,1 billion people with 10% of such protein. Over 53% of the global trade in fish and seafood originates in developing countries whose net trade income (export–import), valued at US$35 billion in 2012, is greater than the net trade income of the other agricultural commodities combined. Furthermore, around 260 million people are involved in global marine capture fisheries, including full-time and part-time jobs in the direct and indirect sectors [67, 81].

As a reference for the economic magnitude of the problems posed by “on land” endocrine disruptor chemicals, according to a series of studies released by the Endocrine Society, and only taking into account medical costs,^25^ routine exposure to EDCs found in pesticides and in every day consumer items in homes costs only to the EU €157 billion annually [82] and $340 billion annually in the US [83], a magnitude similar to the cost of smoking-related illness—the largest single cost coming from effects on children.

## Conclusions—actions needed and potential support by chemical and waste convention

### Urgent measures needed on production and consumption of plastics and waste management

One urgent measure would be a global fully fledged efficient waste collection, management, recycling and environmentally sound disposal systems that would guarantee an almost zero plastic release to the environment. However, this seems a financially challenging and possibly decades-long endeavour. Moreover, while such an infrastructure could be economically feasible in industrial countries, it may not be feasible or cost-effective for developing nations [84]. In addition, the exponentially increasing global trend of plastic production and consumption, in a context of global financial crisis, makes extremely uncertain the ability to achieve already established objectives of reduction of marine litter^26^ at global, regional, sub-regional or national levels. Furthermore, the more frequent and strong flooding events in the different world regions facilitate the flushing of plastic to waters in developing countries but also in industrial countries since plastic waste just get flushed away.

Therefore, urgent and strong actions with relatively low public investment are needed at global level, i.e. policy reforms including extended producer responsibility (EPR) and fiscal and economic instruments. A prevention and ‘Best Available Techniques and Practices’ approach, built on a holistic life cycle basis, could allow scarce resources and effort to be focused on measures that are very likely to reduce the problem by directly attacking the source, similar to the way in which industrial toxic emissions were effectively curbed in some developed countries at the end of the last century, instead of relying on ‘end-of-pipe’ solutions, e.g. focusing only on cleaning measures such as ‘fishing for—floating macro—plastic’, which are not efficient and economically viable in an oceanic scale^27^ and which do not stop the continuous inputs of plastic, the already existing microplastic pollution or sunk plastics or by only assessing and monitoring how much worse the problem it is getting [86].

Although there is still need to carry out focused scientific research to fill the knowledge gaps about the impacts of plastic litter in the marine environment [87], the food chain and human health, the precautionary principle, the already existing scientific evidence and reasonable concerns should be enough to support actions by the scientific, industry, policy and civil society communities to curb the leaking of plastics into the marine environment in the short term. To think in terms of “business as usual” and “adaptation measures” to cope with plastic pollution in the oceans instead of prevention and mitigation measures would lead to another predictable environmental crisis for future generations to cope with. The dangers of working in isolation are already apparent from industry-centred responses such as the development of “oxo-degradable” plastic products, which merely take out of sight plastics by fragmenting them at the end of their lifetime into numerous small but essentially non-degradable pieces [84].

Strong policy actions to curb unnecessary plastic packaging on the demand side on the short term, such as the ban on free single-use plastic bags, or to substantially increase the collection rate of plastic waste, such as the deposit-refund schemes for plastic beverage bottles^28^ which have a demonstrated high rate of success in many countries,^29^ and the ban on plastic microbeads in cosmetics and personal care products, are strongly needed at regional, sub-regional or national levels as part of their strategies for waste management. Initiatives to promote measurement of the types and quantities of plastic used by companies or communities, such as the ‘Plastic Disclosure Project’,^30^ could facilitate accountability and the implementation of measures to reduce avoidable plastic use by the private and public sectors. Designers and producers should avoid creating products that are inherently single use or inevitably destined for landfill [85].

Other measures to consider in developing countries or remote rural communities of Africa, America or Pacific SIDS, with no or few environmentally sound disposal facilities, would be, for example, the take back or repatriation schemes of plastic waste under extended producer responsibility (EPR) schemes, specially for food and beverage plastic packaging, given the clear benefits of plastic versus other packaging materials in reducing the total amount of packaging (in tonnes), as well as the energy required for transportation on the long-haul shipments and the food losses.

Campaigns to make plastic litter socially unacceptable and educate consumers across the supply chain would be necessary elements of any policy of awareness on waste. Designing for recycling would allow to divert important volumes of plastic waste from the waste management systems. It is necessary to work with companies and research institutes, especially in the food sector, to optimize food packaging and materials to avoid unnecessary use of persistent plastics and toxic chemicals. Strong policy actions, as well as more research, development and innovation in green chemistry are needed for the substitution of POPs, EDC and other toxic substances in plastics as well as for the development of more benign alternatives to persistent polymers in the marine environment.

It is important to highlight that compostable bioplastics or plastics labelled as ‘biodegradable in the environment’ are not degraded in marine conditions, where parameters such as temperature, oxygen, and salinity are very different that those expected in a composting process, and so they have equivalent properties in the marine environment in this regard as persistent plastics.^31^ Other innovative materials, such as marine biodegradable polymers, especially for food packaging, could have an important role to play in reducing the environmental damage of plastics leaking to the marine environment, but the biodegradability in marine environment of such alternative plastics (such as the polyhydroxyalkanoates, PHAs) would require further study and validation under a range of conditions in seawater, and internationally accepted certification seals. Further avenues of research on these biomaterials would be to study their complete lifecycle (e.g. to ensure that they do not compete with food production, best options to recycle), potential harms by ingestion to marine biota, and its rate of adsorption of HOC in seawater before its degradation compared with other adsorbing media in the marine environment, including persistent plastics.

Implementing or improving environmentally sound waste collection and management systems of urban waste represents a basic necessary step to reducing plastic inputs, especially in developing economies. Special attention should be paid to avoid creating further environmental and health impacts, for example, by promoting non-best available technology (BAT) waste incineration of plastics without tight environmental controls, which may be an important identified source of POPs, such as dioxins and furans. Effective mandatory or voluntary measures are urgently needed to curb the consumption of single-use plastics, as well as the urgent banning of microplastics in all types of cosmetics and personal care products, even in those countries with 100% coverage of tertiary WWTP.

The actual levels of POPs in marine plastics collected from the sea should be taken into consideration when deciding on management options for marine waste, including recycling.

The implementation of action plans to reduce the input of marine plastic around the world needs to involve all stakeholders from the local and national authorities to international bodies, the scientific community, plastic manufacturers and retailers, tourism and fishing industries, NGOs, etc., to effectively address socio-economic and environmental issues related to plastic pollution from a sustainable and global point of view [89].

### Potential measures suggested in the framework of the Stockholm and Basel Conventions to address marine litter

#### Contribution from the Stockholm Convention on persistent organic pollutants (POPs)

To acknowledge plastic marine litter as an issue of global environmental and health concern, due to its persistence, wide geographical distribution and long-range transport capacity of persistent and toxic chemicals in the marine environment.

Due to the toxic chemical exposure of marine biota through marine plastic litter and the related bioaccumulation and widespread distribution in all marine compartments of persistent micro- and nanoplastics with chemicals of concern acting as persistent organic pollutants in the marine environment, and given the potential human affection to consider:

1. To take into account the risks of additives in plastics with endocrine disruptor properties when selecting and assessing substances for the listing of new POPs in the Stockholm Convention. Some plastic additives with endocrine disruptive properties which might not pass some of the POPs screening criteria such as persistence in water in standard laboratory conditions, are expected to have longer half-life in the plastic due to the protection (or molecular encapsulation) within the polymer matrix, and may have even longer half-life in the marine environment, due to its physical and chemical properties such as lower temperatures, lower oxygen levels, salinity, pH, and lower levels of light in water column and sea floor and sediments, i.e. theoretically “non-persistent” chemical additives or trace monomers in plastics (such as alkylphenols, phthalates, BPA) have been detected in high concentrations in floating polyethylene and polypropylene plastic—the most widely used in packaging—in open oceans [18, 58, 60, 69]. In addition, apart from their mobility and fluxes through all the compartments of the marine environment [15], the new inputs of ‘fresh’ plastic into the marine environment is so continuous and widespread through all the oceans that would be equivalent to the continental or oceanic long-range transport property of highly persistent POPs. Their exposure to marine biota is relevant due to
  - The very low doses of EDCs required to affect the endocrine systems in marine biota and humans [90], compared to those required in toxicological tests to prove carcinogenicity in candidate POPs, especially during the embryo and developing stages,
  - The uptake of microplastics containing those chemicals by marine biota, which may affect biodiversity, food security, food availability and potentially human health, especially if the persistent plastic consumption and production follows the expected growing trends in the coming decades (see Fig. 1), without the necessary environmentally sound waste management and collection facilities being in place globally to avoid plastic leaking into the oceans.
2. The introduction of measures to reduce marine plastic litter in National Implementation Plans for the Stockholm Convention on Persistent Organic Pollutants, such as
  - Promoting substitution and green chemistry to avoid POPs and other harmful chemicals in plastics, especially EDCs.
  - Encouraging plastic waste prevention and supporting development and implementation of safer or more benign alternatives to persistent plastics in the marine environment.
  - Supporting research on environmental and health impacts of marine plastics, microplastics and nanoplastics and related fate of EDCs and POPs.
  - Encouraging ecodesign for better packaging recyclability.
  - Encouraging plastic waste recycling when feasible.
  - Promoting BATs to reduce plastic leakage to oceans and improving information on input loads, sources and originating sectors.
  - Encouraging the improvement and efficiency of collection and sound environmental management of waste.
  - Encouraging changes in consumption and littering behaviour.

#### Contribution from the Basel Convention on hazardous wastes

To acknowledge plastic marine litter as an issue of global environmental and health concern, due to its persistence, wide geographical distribution and long-range transport capacity of toxic chemicals in the marine environment and the need to address it by improvement of waste management and other means.

To consider

1. To include measures to avoid or reduce marine plastic litter in the Strategic Framework for the implementation of the Basel Convention.
2. Revising Annexes I and III of the Convention to ensure the listing of all chemicals with endocrine disruptor substances (EDCs) in plastics that may end up as microplastic waste in the marine environment.
3. The adoption of new guidelines on environmental sound management of plastic and plastic containing wastes, with a view to minimize the possibility of plastic leaks into the oceans coming from waste management.
4. Reviewing policies related to the export of plastic containing waste to countries where no environmentally sound recycling, recovery or final disposal of the plastic materials contained in the waste are guaranteed, i.e. uncontrolled recycling of plastics with toxic chemicals, waste disposal in non-BAT open dumps, or incinerated in cement furnaces with no environmental controls, or non-BAT incinerators without tight environmental measures and controls like dioxin catalyzers, continuous outflow monitoring and sound environmental landfilling of its ashes.
5. Ensuring the best available techniques and best environmental practices are recommended in Basel Convention waste guidelines and manuals to avoid disposal methods that might re-release toxic chemicals into the air, water or soils to safeguard the health of neighbouring communities.
6. Developing efficient strategies for achieving the prevention and minimization of the generation of marine plastic litter.

## Future activities to address marine litter

The Working Group identified a number of possible future activities to address the issue by the Basel and Stockholm Conventions Regional Centres in coordination with existing platforms, or by any other UN Environment institutions, IGOs, governments, NGOs, etc., such as

- Dissemination, information and training activities to improve awareness and knowledge on the risks and challenges posed by marine plastic litter and on measures to combat it.
- Technical assistance and capacity-building activities to support parties and other stakeholders in implementing waste management and efficient waste collection measures to reduce plastic marine litter.
- Develop recommendations to review regional and national regulatory frameworks concerning plastic and plastic containing wastes and inclusion of measures to prevent plastic waste, such as measures to reduce plastic bags consumption and establishment of Deposit and Return schemes for beverage packaging.
- To promote innovation and technology transfer to avoid persistent plastics and sound chemical substitution of toxic components in plastic packaging and other plastics, encouraging plastic waste prevention and supporting development and implementation of safer or more benign alternatives to persistent plastics in the marine environment.
- To assist developing countries, economies in transition and Small Island Developing States with efficient collection and environmentally sound management of plastic waste and plastic packaging, which they are unable to dispose of or recycle in an environmentally sound manner but continue to receive nonetheless, including through take back or repatriation policies under extended producer responsibility (EPR) schemes.

## Footnotes

1 Among the most common polymers found in the marine environment are low density polyethylene (PE-LD), linear low-density polyethylene (PE-LLD), high-density polyethylene (PE-HD), polypropylene (PP), polyethylene terephthalate (PET), polystyrene (PS) and polyvinyl chloride (PVC).

2 The total estimated biomass of fish of 10 g per individual and upwards in the oceans is 529 million tonnes [7], which puts the magnitude of the problem of plastics in the oceans into perspective.

3 Other exogenous causes are natural disasters such as floods, hurricanes and tsunamis.

4 There are other sources of polymers that are not considered in this paper such as cigarette butts; tyre and road wear; and artificial turf infill.

5 This was a strong argument for the law banning microbeads in cosmetics and personal care products in the US in 2015 (Microbead-Free Waters Act).

6 83% of the sampled crustacean *Nephrops norvegicus* in the Sea of Clyde (Scotland) contained plastics (predominately filaments) in their stomachs [14].

7 The study of transformations of matter and energy in systems as they approach equilibrium.

8 The taking in of matter by a living cell by invagination of its membrane.

9 Polymers can also be broken up into monomers by UV radiation, mechanical action, heat and other chemicals [40].

10 Substances that were added intentionally in the virgin polymer and that are incorporated unknowingly or unwillingly when the plastic waste is recycled.

11 Hydrophobicity is a property common to most of the POPs [41].

12 The SIN (Substitute It Now!) List, developed by ChemSec, identifies 32 EDCs of high concern that would require immediate action towards substitution, and 14 more chemicals with ED properties and additional hazardous properties as well. http://chemsec.org/business-tool/sin-list/ (Accessed Mar 2017).

13 A fact that should be taken into account when assessing EDC effects in animal models.

14 TBBPA degrades to Bisphenol A and to TBBPA dimethyl ether. Bisphenol A and phthalates are rapidly metabolised once ingested but their concentration within the tissues varies between species for the same exposure.

15 Short-chained chlorinated paraffins are listed in Annex A (elimination) of the Stockholm Convention since May 2017.

16 PCBs and PCNs have been used to some extent as flame retardants in cables and other polymers including PVC coatings for corrosion protection. Such coatings are sometimes removed from bridges and dams by abrasive blasting and end up in rivers and the sea.

17 http://greensciencepolicy.org/highly-fluorinated-chemicals/.

18 PFCs in the environment can last for millions of years.

19 Marine paint containing tributyltin was forbidden by the International Convention on the Control of Harmful Anti-fouling Systems in Ships (enter into force in 2008), signed by most of the countries.

20 Teuten et al. [22] tested the sorption uptake and desorption kinetics of HOCs in different polymers in laboratory conditions, showing that glassy polymers such as PVC exhibit larger sorption capacities and slower HOC release rates than rubbery polymers such as high-density polyethylene. Mato et al. [59] showed that polyethylene has higher affinity than polypropylene for HOCs.

21 This finding suggests a new non-invasive method, which is to use the PAEs found in plankton as tracers of the exposure/ingestion in cetaceans or other endangered species.

22 Articles with any substance listed under the Stockholm Convention, such as HBCDD used mainly in EPS/XPS polymers, are not allowed to undergo recycling processes, except articles (plastics) with hexa-, hepta-, tetra- or pentabromodiphenyl ethers that would allow some countries to recycle them until 2030, under a exemption of the Convention.

23 For example, 50% of the plastic waste collected for recycling in the EU is exported to third countries with no sound environmental waste management guarantees (source Plastic Recyclers Europe).

24 Precautionary principle by virtue of which where there are threats of serious or irreversible damage, lack of full scientific certainty shall not be used as a reason for postponing cost-effective measures to prevent environmental degradation.

25 The Endocrine Society has recently stated that: “… data reviewed in EDC-2 removes any doubt that EDCs are contributing to increased chronic disease burdens related to obesity, diabetes mellitus, reproduction, thyroid, cancers, and neuroendocrine and neurodevelopmental functions” [45].

26 The Honolulu Strategy, the global framework to prevent marine litter, does not prescribe specific marine debris reduction targets but expects “substantial progress” by 2030. The UN’s Sustainable Development Goal number 14 (Sustainable Oceans) aims to “prevent and significantly reduce” marine litter in 2025. In the European Union, a 30% reduction for beach litter by 2025, compared with 2015 levels, has been proposed for all its regional seas.

27 Clean-up may be a suitable last resort for addressing marine litter in limited zones such as urban areas, tourist beaches and ports where the litter causes severe social and economical damage [85], or in marine special protected areas (SPAs).

28 Plastic beverage bottles represent around 20% of all plastic packaging waste in the EU.

29 Compared with the relatively low and stagnate rates of curbside separate collection of plastic packaging waste, with the added benefit of delivering a high-quality product ready for recycling [88].

30 http://Plasticdisclosure.org.

31 Biodegradation according to EN13432 is considered to be complete if at least 90% of the material has been converted into carbon dioxide (the remainder is due to the fact that besides carbon dioxide, water and biomass are produced during biodegradation).When all the organic carbons in the polymer are converted, it is referred as complete mineralisation.
